# Correction: Transcriptional profiling at whole population and single cell levels reveals somatosensory neuron molecular diversity

**DOI:** 10.7554/eLife.06720

**Published:** 2015-02-06

**Authors:** Isaac M Chiu, Lee B Barrett, Erika K Williams, David E Strochlic, Seungkyu Lee, Andy D Weyer, Shan Lou, Gregory Bryman, David P Roberson, Nader Ghasemlou, Cara Piccoli, Ezgi Ahat, Victor Wang, Enrique J Cobos, Cheryl L Stucky, Qiufu Ma, Stephen D Liberles, Clifford Woolf

Chiu IM, Barrett LB, Williams EK, Strochlic DE, Lee S, Weyer AD, Lou S, Bryman G, Roberson DP, Ghasemlou N, Piccoli C, Ahat E, Wang V, Cobos EH, Stucky CL, Ma Q, Liberles SD, Woolf C. 2014. Transcriptional profiling at whole population and single cell levels reveals somatosensory neuron molecular diversity. *eLife* **3**:04660. doi: 10.7554/eLife.04660

Published 19 December 2014

Supplementary file 1 was missing values and the FDR values shown in Column K were incorrect as a result.

Supplementary file 1 has been corrected accordingly.

